# Case Report: A Case of COVID Vaccine-Induced Thrombotic Thrombocytopenia Manifested as Pulmonary Embolism and Hemorrhagia. A First Reported Case From Slovakia

**DOI:** 10.3389/fmed.2021.789972

**Published:** 2022-01-04

**Authors:** Martin Ihnatko, Ivana Truchla, L'udmila Ihnatková, Zoltán Prohászka, Ivica Lazúrová

**Affiliations:** ^1^First Department of Internal Medicine, Faculty of Medicine, Pavol Jozef Šafárik University in Košice, Košice, Slovakia; ^2^Department of Hematology and Oncohematology, Faculty of Medicine, Pavol Jozef Šafárik University in Košice, Košice, Slovakia; ^3^Research Laboratory, Department of Internal Medicine and Hematology, Semmelweis University, Budapest, Hungary

**Keywords:** COVID-19, vaccine-induced thrombotic thrombocytopenia, pulmonary embolism, adenoviral vector vaccine, hemorrhagia

## Abstract

COVID-19 vaccine-induced thrombotic thrombocytopenia (VITT) is a rare complication of adenoviral vector (ChAdOx1 nCoV-19) vaccine administration. It is presented as thrombocytopenia and thrombotic manifestations in various sites, especially in cerebral veins. Pulmonary emboli have been reported rarely. We present a case of a young male patient who developed severe thrombocytopenia and pulmonary embolism 12 days after the first dose of the vaccine. Severe thrombocytopenia, skin hematomas, and segmental pulmonary emboli were detected. Anti-platelet factor 4 (aPF-4) antibody was highly positive supporting the diagnosis of VITT. Prompt treatment with fondaparinux, intravenous immunoglobulin, and prednisone led to a marked improvement of clinical condition and thrombocytes count. We report the first known case of VITT in Slovakia.

## Introduction

COVID-19 is a global pandemic disease with a high morbidity and mortality and a deleterious impact on the human population. To date, more than 4.6 million people globally have died from COVID-19 (1). Hence, the massive vaccination efforts represent the only way to mitigate the negative consequences of this pandemic. Several vaccines have been developed to date, with various common and rare adverse effects (2).

It is known that the overall risk of thrombosis and thromboembolic complications due to COVID-19 is increased (3, 4). On the other hand, there are reports on thrombotic complications after vaccination against this infection, especially following the administration of adenovirus-based vaccines. Since March 2021, several cases of uncommon vaccine-related thrombotic events associated with thrombocytopenia have been reported, in particular after the ChAdOx1 nCov-19 vaccine administration (5–8). These complications have been termed vaccine-induced thrombotic thrombocytopenia (VITT), vaccine-induced prothrombotic immune thrombocytopenia (VIPIT), or vaccine associated thrombotic thrombocytopenia (VATT) (9). This severe condition is characterized by unusual location of thrombosis, mostly in cerebral veins, however other sites of thrombosis or pulmonary embolism have been published. The mortality rate of VITT is high, reaching up to 25% (7).

The overall risk for VITT development following administration of the AstraZeneca vaccine is low (10.9 cases per million doses). To date, several hundreds of cases have been reported. Incidence of pulmonary embolism occurs in 0.08 people per million and it is more common in patients over 65 years (10).

In this report we refer to the first known case of a young patient with VITT manifested by severe thrombocytopenia, skin hematomas, and pulmonary embolism in Slovakia.

## Case Presentation

A 31-year-old man was admitted to the emergency unit because of multiple skin hematomas and severe thrombocytopenia. His family history as well as previous medical history were unremarkable and insignificant regarding venous thromboembolism. The patient did not have a history of autoimmune disease or COVID-19 infection.

In April 2021, the patient was vaccinated with the first dose of ChAdOx1 nCov-19 vaccine. After vaccination he developed fever and headache that disappeared within 2 days. On the seventh day after vaccination, his headache returned and on the twelfth day he presented skin hemorrhage and mild transient dyspnea. Because of its progression and laboratory finding of severe thrombocytopenia, he was referred to the hospital.

**Figure 1:**
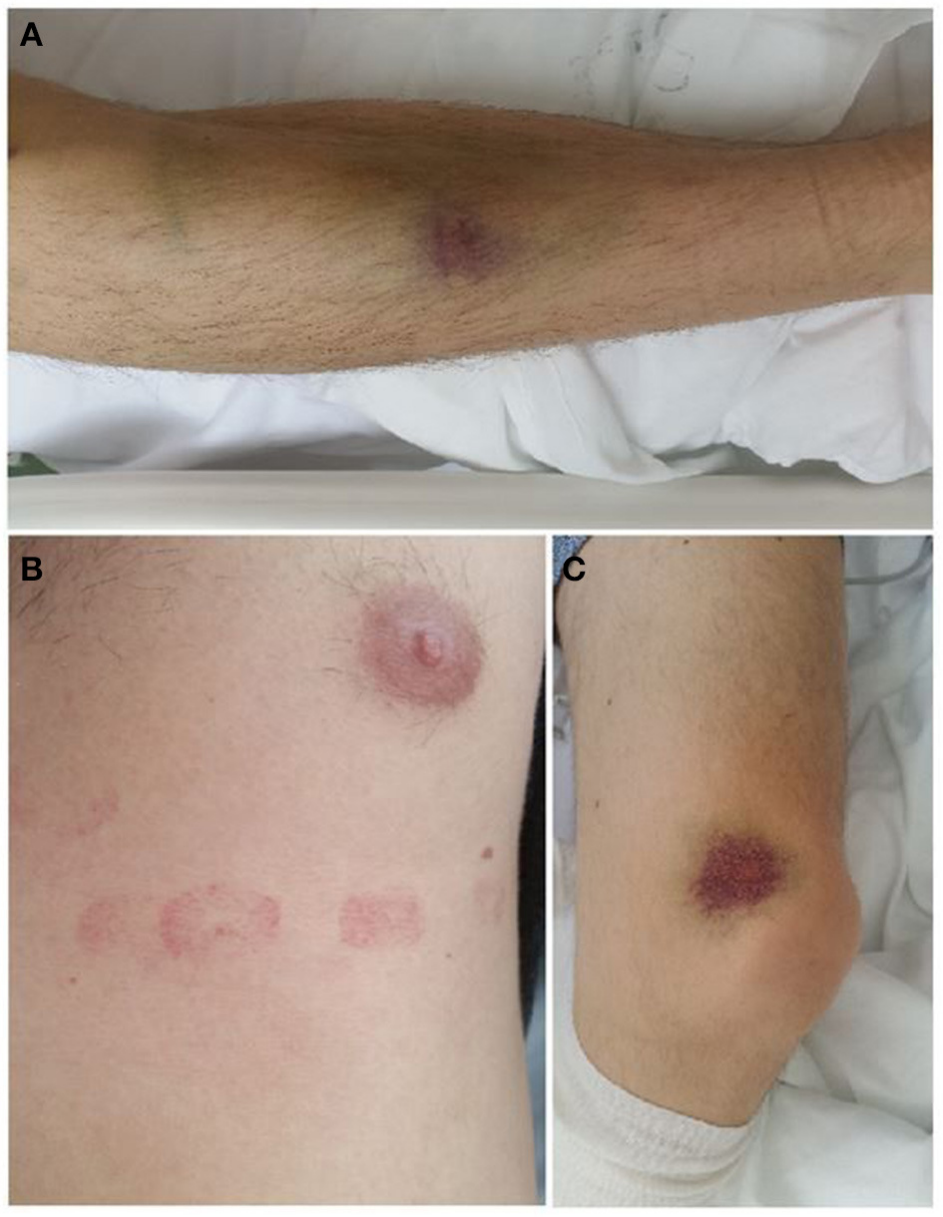
Skin hematomas on the chest wall and lower extremities. **(A)** Right foreleg **(B)** Left side of the chest wall **(C)** Medial side of the left knee joint.

On physical examination, the patient was afebrile, eupneic, and with multiple skin hematomas on the chest wall and both lower extremities (Figure 1). No petechias were found. His blood pressure was 155/95 mmHg, heart rate was 96/min, and oxygen saturation value was normal (96%).

### Laboratory Investigations

Laboratory investigations revealed severe thrombocytopenia of 25 x 10^9^/L (normal 150-400 x 10^9^/L) whereas erythrocytes and leukocytes count were in normal range. Serum D dimer concentration was markedly elevated 39.198 mg/L (normal 0.03-0.5 mg/L) while serum fibrinogen concentration was slightly decreased 0.87 g/L (normal 1.8-3.5 g/L). Prothrombin time (PT) and activated partial thromboplastin time (APTT) were not significantly changed.

### Biochemical Evaluation

Biochemical evaluation demonstrated mild hyperbilirubinemia 41.3 umol/L (normal 5-21 umol/L) and slightly elevated hepatic enzymes, i.e., AST 0.98 ukat/L (normal 0.05-0.85 ukat/L), ALT 1.54 ukat/L (normal 0.05-0.85 ukat/L), and GMT 1.37 ukat/L (normal 0.05-0.92 ukat/L).

### Immunological Evaluation

Anti-platelet factor 4 IgG (aPF4G) (Antibodies-Online Cat# ABIN351496, RRID:AB_10825453) measured by AESKULISA HIT II Kit was highly positive with the serum concentration of 38.67 U/ml (ref: <12 U/ml), supporting the diagnosis of vaccine-induced thrombotic thrombocytopenia (VITT). RT-PCR test for the presence of SARS-Cov-2 was negative at the time of admission. SARS-CoV-2 Total antibodies were not detected (0.105, normal cutoff index 0.000-0.999) and SARS-CoV-2 spike protein (S) antibodies (Leinco Technologies Cat# LT3500, RRID:AB_2893953) were elevated (32.38 U/ml, normal 0.00-0.8 U/ml).

To rule out other possible causes of thrombotic thrombocytopenia, we also measured ADAMTS13 (MGI Cat# 3708874, RRID:MGI:3708874), which was found to be in normal range (Table 1). Results of immunological evaluation are shown in Table 1. Complement profile did not show signs of dysregulation or consumption. Slight elevation of terminal pathway activation marker was found.

**Table 1:** Results of immunological evaluation in the patient with VITT.

**Parameter**	**Result**	**Reference range**
Anti-platelet factor 4 IgG (aPF4G)	38.67 U/ml	<12
ADAMTS13 metalloprotease activity	97%	67–150
Total complement activity classical pathway (hemolytic test)	87 CH50/ml	48–103
Total complement activity alternative pathway (WIELISA-Alt)	111%	70–125
Complement C3	1.21 g/L	0.9–1.8
Complement C4	0.44 g/L	0.15–0.55
Factor H antigen	923 mg/L	250–880
Complement factor I antigen	134%	70–130 %
Complement factor B antigen	185%	70–130
Anti-factor H IgG autoantibody (Millipore Cat# 341276-1ML, RRID:AB_211828)	76 AU/ml	<110
C1q antigen	111 mg/L	60–180
Anti-C1q IgG autoantibody (Creative Diagnostics Cat# DPABH-16000, RRID:AB_2515874)	3 U/ml	<52
sC5b-9 (terminal complement complex)	335 ng/ml	110–252
Haptoglobin	2.08 g/L	0.3–2.0

### Imaging Methods

X ray of the chest did not detect any pathological changes, while abdominal ultrasound demonstrated cholecystolithiasis with small stone inside gallbladder. On echocardiography there were no significant changes in cardiac morphology or function. No signs of right ventricle hypertrophy or dilation were found. Ejection fraction was 65%. Due to mild dyspnea in previous medical history and markedly elevated serum D dimer concentration, CT pulmonary angiography was performed. It revealed an emboli in the segmental pulmonary arteries bilaterally. CT cerebral venography did not demonstrate cerebral venous thrombosis and Doppler ultrasound of both legs did not detect deep vein thrombosis of peripheral leg veins.

Based on these results we assumed the diagnosis was a vaccine-induced immune thrombotic thrombocytopenia and we initiated treatment with subcutaneous fondaparinux 2.5-7.5 mg daily, intravenous immunoglobulin (IVIG) of dose 1 g/kg, and prednisone 1 mg/kg per os. Within a few days after treatment, platelet count rapidly increased up to 103 x 10^9^/L and serum D dimer concentration decreased to 1.34 mg/L. Clinical condition of the patient rapidly improved and we registered regression of skin hematomas. On the ninth day of hospitalization, the patient was discharged from the hospital.

## Discussion

In this report, we describe the first case of VITT identified and demonstrated in the Slovak Republic. VITT is a serious complication of ChAdOx1 nCoV-19 vaccine. Clinically it mimics autoimmune heparin-induced thrombocytopenia (HIT), which also presents with raised anti-PF4-antibodies (2, 11). Data indicate that the vaccine triggers formation of anti-platelet factor 4 (anti-PF4) immunoglobulin G which activates platelet aggregation resulting in a clinical manifestation of thrombosis and thrombocytes consumption (12, 13). Another possible mechanism includes adenoviral vector entry into megakaryocytes with the subsequent expression of spike protein on platelet surfaces leading to platelet activation by the vector (10). The positivity of anti-PF4 antibody in our patient favors the proposed autoimmune mechanism.

VITT manifests most often with unusual thromboses, but sometimes also with usual thromboses, like deep vein thrombosis of legs or pulmonary embolism (PE). Thrombotic manifestations of VITT were reported in various sites, such as cerebral, abdominal, i.e., splenic, renal, and hepatic veins (6–8, 14). Cerebral venous thrombosis is probably the most common site of thrombosis and occurs in 38-80% of reported cases (9).

Severe thrombocytopenia becomes clinically evident usually within 5-30 days after vaccine administration. In the study of Pavord et al., authors identified 170 definite cases of VITT, all from patients who received the first dose of ChAdOx1 nCoV-19 vaccine. Clinical manifestation presented 5-48 days after vaccination. There was no sex preponderance and known or detectable risk factors. Overall mortality was 22%, and was the highest in patients with intracranial bleeding (15).

In our patient, the first symptoms of skin bleeding appeared approximately on the twelfth day after receiving the ChAdOx1 nCoV-19 vaccine. Except for tachycardia, he did not present any other symptoms of thromboembolic disease. Laboratory findings, i.e., thrombocytopenia, increased serum D dimer concentration, lower fibrinogen concentration, and positive anti-PF4 antibodies were typical for VITT diagnosis. CT pulmonary angiography was realized with positive finding of pulmonary embolism in segmental pulmonary arteries bilaterally. Other investigations did not reveal thrombosis as the source of pulmonary embolism. Therefore, we assumed the primary pulmonary thrombosis should also be considered in our patient. This situation has been described in patients with COVID-19 disease (16–18).

Treatment of VITT should be managed like HIT syndrome and should be started immediately. High dose IVIG should be administered with non-heparin antithrombotic treatment by anticoagulant (fondaparinux, danaparoid, or argatroban). Glucocorticoids and plasma exchange are also therapeutic options to reduce anti-PF4 antibodies levels (10, 19–21). Platelet transfusion is contraindicated and can be considered only in severe bleeding complications (22). Timeline of patient's results initially and after treatment is shown in Table 2.

**Table 2:** Timeline of patient's results.

**Laboratory test result** **Reference range**	**Initial**	**After treatment**
Platelets (150–400 ×10^9^/L)	25	103
D dimer (0.03–0.5 mg/L)	39.198	1.34
Fibrinogen (1.8–3.5 g/L)	0.87	1.10
Prothrombin time (75–125%)	73	94
Activated partial thromboplastin time (26–38 s)	40.6	28.7
C-reactive protein (0.1–5.0 mg/L)	9.31	0.45

## Conclusion

This case report highlights the potentially life-threatening complication associated with ChAdOx1 nCoV-19 vaccine. Data from the last 6 months showed that VITT may be more frequent than has been reported in previous studies. There are still some uncertainties about this pathologic condition and future studies could help us to identify prognostic markers for VITT and to improve outcomes of the disease.

## Data Availability Statement

The original contributions presented in the study are included in the article/supplementary material, further inquiries can be directed to the corresponding author/s.

## Ethics Statement

Written informed consent was obtained from the individual(s) for the publication of any potentially identifiable images or data included in this article.

## Author Contributions

IL: conception and design of the work. MI, IT, and ZP: substantial contributions to the acquisition of data for the work. MI, IT, L'I, ZP, and IL: substantial contributions to the analysis of data for the work and interpretation of data for the work. IL, MI, and L'I: drafting the work. IL and ZP: revising the draft of the work critically for important intellectual content and final approval of the version to be published. All authors approved the final version.

## Funding

This work was supported by the grants from National Office for Innovation and Research (2020-1.1.6-JOVO-2021-00013) for the research in the laboratory of ZP.

## Conflict of Interest

The authors declare that the research was conducted in the absence of any commercial or financial relationships that could be construed as a potential conflict of interest.

## Publisher's Note

All claims expressed in this article are solely those of the authors and do not necessarily represent those of their affiliated organizations, or those of the publisher, the editors and the reviewers. Any product that may be evaluated in this article, or claim that may be made by its manufacturer, is not guaranteed or endorsed by the publisher.
